# 

**Published:** 1860-11

**Authors:** 


### DENTAL REGISTER

### OF THE WEST.

EDITED BY

J. TAFT AND GEO. WATT.

NOVEMBER–1860.

MONTHLY:
Three Dollars per Annum, in advance.

CINCINNATI:

J. T. TOLAND, PUBLISHER AND PROPRIETOR.

J. TAFT, DENTIST, No. 56 WEST FOURTH STREET.

